# Supplementary material for: Metabolomic Analysis of Germinated Brown Rice at Different Germination Stages
Source: Foods. 2020 Aug 17;9(8):1130. doi: 10.3390/foods9081130 (PMC7491196; doi:10.3390/foods9081130)
Supplement: Supplementary file 1 [file foods-09-01130-s001.pdf]

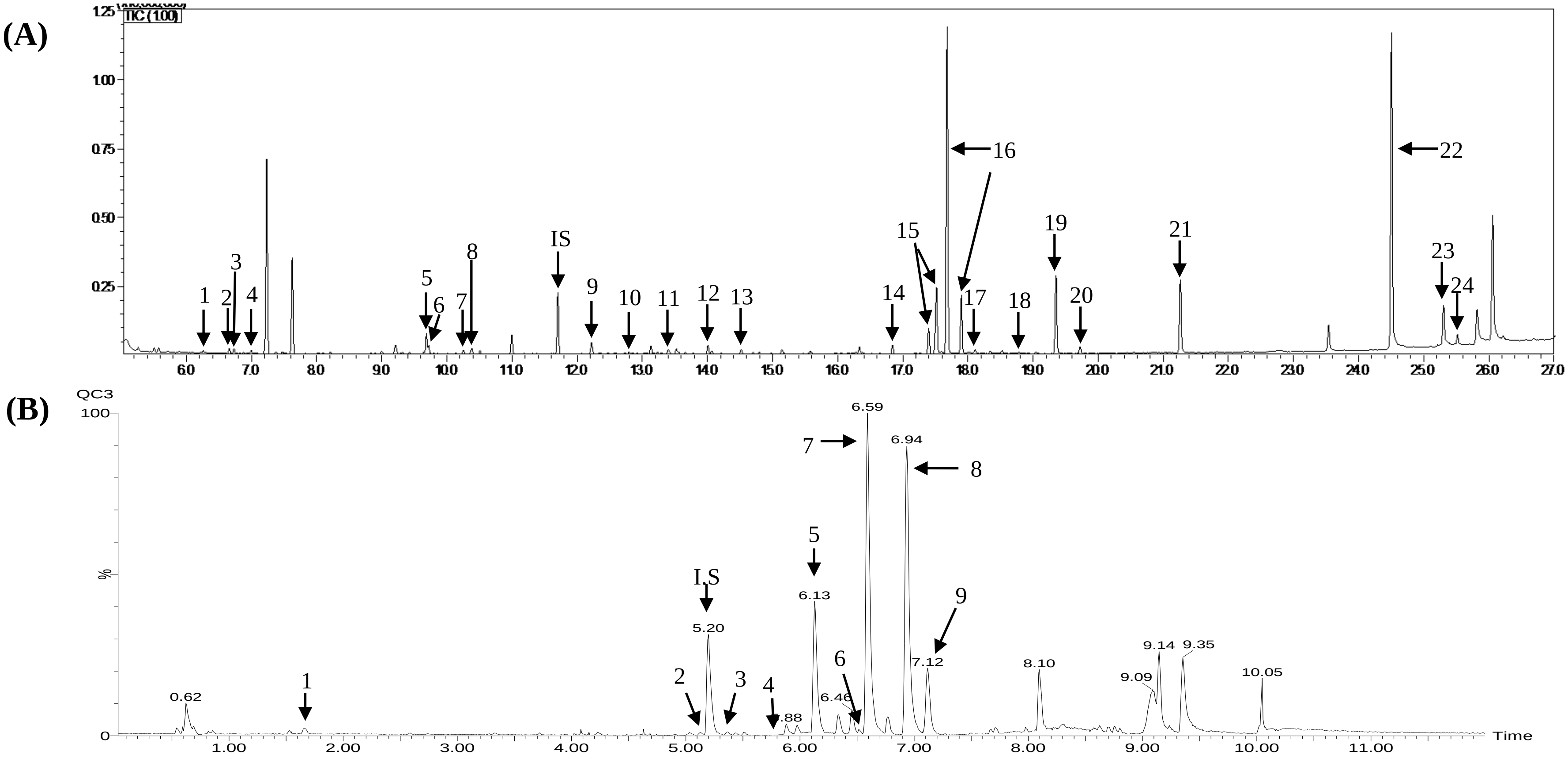

Supplementary Figure 1. Representative (A) gas chromatogram and (B) liquid chromatogram of the rice extract. Rice extracted with 50% methanol was analyzed by GC-MS equipped with a DB-5ms capillary column and UPLC-Q-TOF MS equipped with Acquity UPLC BEH C18 column in positive electrospray ionization mode. The identified metabolites are numbered as follows. For GC-MS: 1, lactic acid; 2, perhydrogeraniol; 3, 4,8-dimethylnonanol; 4, alanine; 5, phosphoric acid; 6, glycerol; 7, 2-isopropyl-5-methyl-1-heptanol; 8, 2,4-diethyl-1-heptanol; 9, 1,4-diacetylbenzene; 10, malic acid; 11, γ-aminobutyric acid (GABA); 12, p-hydroxydiisopropylbenzene; 13, glutamic acid; 14, citric acid; 15, fructose; 16, glucose; 17, sorbitol; 18, gluconic acid; 19, palmitic acid; 20, myo-inositol; 21, stearic acid; 22, sucrose; 23, cellobiose; 24, maltose. For LC-MS: 1, tryptophan; 2, palmitic acid hydrazide; 3, PC(16:0/2:0); 4, phytosphingosine; 5, LPC(14:0); 6, LPE(18:2); 7, LPC(18:2); 8, LPC(16:0); 9, LPC(18:1).
